# Supplementary material for: Untargeted Metabolomics of Slc13a5 Deficiency Reveal Critical Liver–Brain Axis for Lipid Homeostasis
Source: Metabolites. 2022 Apr 14;12(4):351. doi: 10.3390/metabo12040351 (PMC9032242; doi:10.3390/metabo12040351)
Supplement: Supplementary file 1 [file metabolites-12-00351-s001.zip › metabolites-1641645-supplementary.pdf]

Supplementary Data

Untargeted Metabolomics of Slc13a5 Deficiency Reveal Critical Liver-Brain Axis for Lipid Homeostasis

Sofia Milosavljevic[1,2], Kevin E. Glinton[1], Xiqi Li[1], Cláudia Medeiros[3], Patrick Gillespie[3], John R. Seavitt[1], Brett H. Graham[3], and Sarah H. Elsea[1,*]

[1]Department of Molecular and Human Genetics, Baylor College of Medicine, Houston, TX,USA
[2]Harvard Medical School, Boston, MA, USA
[3]Department of Medical and Molecular Genetics, Indiana University School of Medicine, Indianapolis, IN, USA *Correspondence: sarah.elsea@bcm.edu; Tel.: +1-713-798-5484

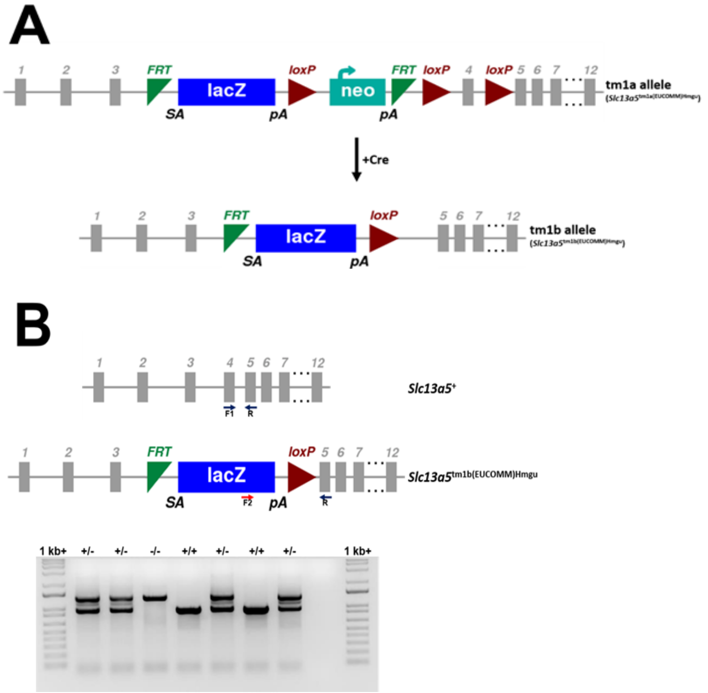

Figure S1. Generation and Genotyping of Slc13a5 Deficiency Mice. A. EUCOMM production of mouse model of Slc13a5 deficiency (C57BL/6N-$Slc13a5^{tm1b(EUCOMM)Hmgu}$/*Ieg*). The *tm1a* allele has a *lacZ* gene trap cassette and a neomycin resistant transgene flanked by indicated FRT and loxP sites inserted in the intron between exons 3 and 4 of *Slc13a5*, as well as an additional *loxP* site inserted in the intron between exons 4 and 5. The $Slc13a5^{tm1b}$ mutant allele is generated by Cre-mediated excision of *Slc13a5* exon 4 and the *neo* cassette from the $Slc13a5^{tm1a}$ allele, which results in a frameshift mutation, leaving the *lacZ* gene trap intact. Partial genomic maps of $Slc13a5^{tm1a}$ and $Slc13a5^{tm1b}$ alleles are depicted. **B**. Genotyping of Slc13a5-deficient mice (C57BL/6N-$Slc13a5^{tm1b(EUCOMM)Hmgu}$/*Ieg*). The wild type allele PCR amplicon is 919 bp, and the mutant allele PCR amplicon is 1301 bp. Partial genomic maps of $Slc13a5^{+}$ and $Slc13a5^{tm1b}$ alleles with indicated PCR primer binding sites, as well as a representative picture of agarose gel electrophoretic analysis of PCR genotyping results, are shown. "1 kb+" = 1 kb Plus DNA ladder. "+/+" = $Slc13a5^{+/+}$; "+/-" = $Slc13a5^{+/tm1b}$; and "-/-" = $Slc13a5^{tm1b/tm1b}$ [1].

1. Skarnes WC, Rosen B, West AP, Koutsourakis M, Bushell W, Iyer V, Mujica AO, Thomas M, Harrow J, Cox T, et al. A conditional knockout resource for the genome-wide study of mouse gene function. *Nature*, 2011. **474**(7351): p. 337-42.

Online eTable S1. Slc13a5 Deficiency Mouse Tissue Metabolite Flux. Interactive table: https://app.powerbi.com/view?r=eyJrIjoiN2I4YTM3ZjMtZGE1MS00ODk0LThlNmItYWIzY2IzZjY2ZGE1IiwidCI6IjZiYmUyYmZmLWNjMzQtNDBlYi05OTZmLWZiNGY2ZjI5MTdiZiIsImMiOjl9
